# Weak Eyes

**Published:** 1860-01

**Authors:** 


### WEAK EYES.

MANY who are troubled with weak eyes, by avoiding the use of them in reading, sewing, and the like, until after breakfast, will be able to use them with greater comfort for the remainder of the day, the reason being, that in the digestion of the food the blood is called in from all parts of the system, to a certain extent, to aid the stomach in that important process; besides, the food eaten gives general strength, imparts a stimulus to the whole man, and the eyes partake of their share.

